# Adjuvant-dependent effects on the safety and efficacy of inactivated SARS-CoV-2 vaccines during heterologous infection by a SARS-related coronavirus

**DOI:** 10.21203/rs.3.rs-3401539/v1

**Published:** 2023-10-27

**Authors:** Mark Heise, Jacob Dillard, Sharon Taft-Benz, Audrey Knight, Elizabeth Anderson, Katia Pressey, Breantié Parotti, Sabian Martinez, Jennifer Diaz, Sanjay Sarkar, Emily Madden, Gabriela De la Cruz, Lily Adams, Kenneth Dinnon, Sarah Leist, David Martinez, Alexandra Schaefer, John Powers, Boyd Yount, Izabella Castillo, Noah Morales, Jane Burdick, Mia Katrina Evangelista, Lauren Ralph, Nicholas Pankow, Colton Linnertz, Prem Lakshmanane, Stephanie Montgomery, Martin Ferris, Ralph Baric, Victoria Baxter

**Affiliations:** University of North Carolina at Chapel Hill; University of North Carolina at Chapel Hill; University of North Carolina; Dallas Tissue Research; University of North Carolina at Chapel HIll; University of North Carolina at Chapel Hill; University of North Carolina at Chapel Hill; University of North Carolina at Chapel Hill; University of North Carolina at Chapel Hill; Southern Research; EpiCypher; University of North Carolina at Chapel Hill; Gryphon Scientific LLC; The Rockefeller University; University of North Carolina at Chapel Hill; Yale University; University of North Carolina at Chapel Hill; University of North Carolina at Chapel Hill; Department of Epidemiology, Gillings School of Public Health, University of North Carolina at Chapel Hill; University of North Carolina at Chapel Hill; University of North Carolina at Chapel Hill; University of North Carolina at Chapel Hill; University of North Carolina at Chapel Hill; University of North Carolina at Chapel Hill; University of North Carolina at Chapel Hill; University of North Carolina at Chapel Hill; Department of Microbiology and Immunology, University of North Carolina School of Medicine, Chapel Hill, NC; University of North Carolina at Chapel Hill; University of North Carolina; University of North Carolina at Chapel Hill; Texas Biomedical Research Institute, San Antonio, Texas, USA

## Abstract

Inactivated whole virus SARS-CoV-2 vaccines adjuvanted with aluminum hydroxide (Alum) are among the most widely used COVID-19 vaccines globally and have been critical to the COVID-19 pandemic response. Although these vaccines are protective against homologous virus infection in healthy recipients, the emergence of novel SARS-CoV-2 variants and the presence of large zoonotic reservoirs provide significant opportunities for vaccine breakthrough, which raises the risk of adverse outcomes including vaccine-associated enhanced respiratory disease (VAERD). To evaluate this possibility, we tested the performance of an inactivated SARS-CoV-2 vaccine (iCoV2) in combination with Alum against either homologous or heterologous coronavirus challenge in a mouse model of coronavirus-induced pulmonary disease. Consistent with human results, iCoV2 + Alum protected against homologous challenge. However, challenge with a heterologous SARS-related coronavirus, Rs-SHC014-CoV (SHC014), up to at least 10 months post-vaccination, resulted in VAERD in iCoV2 + Alum-vaccinated animals, characterized by pulmonary eosinophilic infiltrates, enhanced pulmonary pathology, delayed viral clearance, and decreased pulmonary function. In contrast, vaccination with iCoV2 in combination with an alternative adjuvant (RIBI) did not induce VAERD and promoted enhanced SHC014 clearance. Further characterization of iCoV2 + Alum-induced immunity suggested that CD4^+^ T cells were a major driver of VAERD, and these responses were partially reversed by re-boosting with recombinant Spike protein + RIBI adjuvant. These results highlight potential risks associated with vaccine breakthrough in recipients of Alum-adjuvanted inactivated vaccines and provide important insights into factors affecting both the safety and efficacy of coronavirus vaccines in the face of heterologous virus infections.

## INTRODUCTION

SARS-CoV-2 has caused a global public health crisis resulting in nearly 7 million confirmed deaths and greater than 10 trillion dollars in economic losses [1, 2]. In addition to the ongoing challenges caused by SARS-CoV-2, SARS-related coronaviruses (SARS-r-CoVs) continue to represent a major pandemic threat. Emergence events by zoonotic coronaviruses have occurred at least seven times throughout human history, with three highly pathogenic coronaviruses entering the human population from 2003 to 2019 alone [3]. SARS-r-CoVs continue to circulate in zoonotic reservoirs and threaten to cause human infections [3–5]. Given the frequency of recent emergence events and the continuing threat posed by circulating SARS-r-CoVs, future zoonotic SARS-r-CoV epidemics are likely.

In the context of COVID-19, there has been an unprecedented effort devoted to the development, testing and deployment of SARS-CoV-2 vaccines. To date, over 13.3 billion vaccine doses have been administered worldwide [6]. Among other important vaccine platforms, whole virus-based inactivated vaccines have had a major global impact on the COVID-19 pandemic. Inactivated vaccines are relatively simple to produce, lack special storage requirements, and are safe for immunocompromised individuals, making these vaccines attractive for widespread use [7]. Inactivated vaccines administered with the aluminum hydroxide adjuvant (Alum) accounted for approximately half of all COVID-19 vaccines (nearly 5 billion doses) administered by 2022 [8]. Three inactivated COVID-19 vaccines (developed by Sinovac, Sinopharm and Bharat Biotech) are approved for emergency use by the World Health Organization [9].

Inactivated vaccines provide moderate protection against symptomatic infection with significant and sustainable protection against severe disease and mortality [7, 8, 10–16]. However, neutralizing antibody titers induced by inactivated vaccines wane relatively quickly, and these vaccines are not highly effective against variants of concern (VOC) like B.1.617.2 (Delta) and Omicron subvariants [7, 8, 12, 17]. This raises concerns about breakthrough infections in vaccinated individuals, particularly in individuals who do not mount strong immune responses to vaccines, including those 65 years of age and older.

Such breakthrough infections due to vaccine failure are sometimes associated with vaccine-associated enhanced respiratory disease (VAERD), an outcome that has been observed historically with the formalin-inactivated respiratory syncytial virus (RSV) and formalin-inactivated measles virus vaccines [18–27]. Vaccine-induced pathology was reported in several preclinical studies of SARS-CoV and MERS-CoV vaccines, including inactivated vaccines, replicon-vectored vaccines, and recombinant subunit Spike protein vaccines [21, 28–36]. Vaccine-induced pathology following homologous or heterologous viral infection was characterized by type 2 immunopathology, including pulmonary eosinophil infiltration and upregulation of type 2 cytokines. The majority of these studies were performed in BALB/c mice; however, vaccine-enhanced immunopathology was also reported in C57BL/6 mice, ferrets and non-human primates. DiPiazza and Leist et al reported that inactivated SARS-CoV-2 vaccines induce suboptimal immune responses, including weak neutralizing antibodies, and can cause type 2-associated immunopathology in multiple strains of mice following SARS-CoV-2 infection [37]. More recently, Ebenig et al reported type 2 immunopathology in SARS-CoV-2-infected hamsters that were previously vaccinated with a suboptimal Spike protein vaccine [38].

Given the rising threat of vaccine breakthrough by VOC and the potential for future SARS-r-CoV epidemics, combined with the risk of VAERD in the context of vaccine failure, we evaluated the safety and efficacy of an inactivated SARS-CoV-2 vaccine using established mouse models of homologous and heterologous SARS-r-CoV respiratory infection. Our results demonstrate that when administered with Alum, an inactivated SARS-CoV-2 vaccine (iCoV2 + Alum) protects against infection by homologous virus and a VOC (B.1.351), but results in type 2 VAERD, including enhanced clinical disease, in mice during infection by a heterologous virus, Rs-SHC014-CoV (SHC014). iCoV2 + Alum vaccination also fails to control SHC014 replication and causes delayed SHC014 clearance and impairs respiratory function in a subset of vaccinated animals. However, when administered with an alternative adjuvant (RIBI, Sigma Adjuvant System) reported to induce type 1 immune responses [39, 40], iCoV2 cross-protects against SHC014 without causing VAERD. We also observed that secondary boost vaccination with a heterologous vaccine lacking Alum partially reduces VAERD. Lastly, we find that CD4^+^ T cells play a major role in driving VAERD. In summary, our findings (i) indicate that the safety and efficacy of an inactivated SARS-CoV-2 vaccine during heterologous SARS-r-CoV infection are adjuvant-dependent, (ii) demonstrate the utility of evaluating coronavirus vaccines against heterologous viruses to identify potential safety issues during preclinical development, (iii) underscore the need to preempt vaccine breakthrough and VAERD with universal coronavirus vaccines, and (iv) suggest the need for increased surveillance to identify VAERD in humans receiving Alum-based vaccines.

## RESULTS

### Inactivated SARS-CoV-2 vaccine protects against homologous SARS-CoV-2 in mice but causes adjuvant-dependent type 2 inflammation

**Figure 1:**
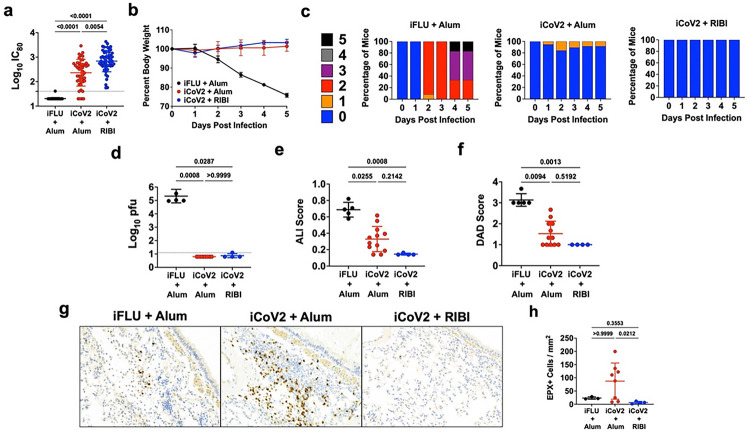
Inactivated SARS-CoV-2 + aluminum hydroxide (iCoV2 + Alum) vaccination protects mice from pathogenic SARS-CoV-2 challenge but causes adjuvant-dependent type 2 inflammation. Mice were vaccinated with inactivated SARS-CoV-2 formulated with either Alum or RIBI adjuvants and challenged 4 weeks post-boost with SARS-CoV-2 MA10. Pulmonary tissue specimens were collected at 5 DPI. **(a)** Post-boost serum samples were collected prior to challenge and neutralizing antibody titers against SARS-CoV-2 (D614G) were measured using a luminescence-based microneutralization assay. Log-transformed results reported as IC_80_. Combined results from 4 independent replicates.**(b)** Body weights were measured daily and reported as percent relative to baseline. **(c)** Clinical disease was evaluated daily using the clinical scoring system below. **(d)** Pulmonary viral titers were quantified by plaque assay and log-transformed results reported as log_10_ pfu. **(e, f)** H&E-stained pulmonary specimens were analyzed by a blinded pathologist to evaluate ALI (e) and DAD (f). **(g, h)** Pulmonary eosinophils (EPX^+^ cells) were measured using immunohistochemical staining (representative micrographs, 100X magnification in g) and quantified (h). **(a, d-f, h)** Individual data points represent biological replicates. Results analyzed by Kruskal-Wallis test with Dunn’s multiple comparisons correction (alpha = 0.05). Solid horizontal lines overlaying data represent group means. Error bars represent group SD. Dotted lines represent assay LOD. Solid horizontal lines above data represent pairwise comparisons with P values. Results from 2 (e, f, h), 3 (d), or 4 (a) independent replicates (effects reproduced in each replicate). **(b)** Reported as group mean ± SD. Analyzed by two-way ANOVA with Tukey’s multiple comparisons. **(b, c)** Representative results from a single experiment. Number of animals per group: iFLU + Alum (12), iCoV2 + Alum (19), iCoV2 + RIBI (4). Clinical scoring system: 0 = normal (blue), 1 = piloerection (orange), 2 = piloerection + kyphosis (red), 3 = piloerection, kyphosis and reduced movement (purple), 4 = markedly reduced movement and/or labored breathing (gray) and 5 = moribund, dead or euthanized (black). iFLU = inactivated influenza virus vaccine; iCoV2 = inactivated SARS-CoV-2 vaccine; Alum = aluminum hydroxide adjuvant; RIBI = Sigma Adjuvant System adjuvant; DPI = days post-infection; IC_80_ = 80% serum reciprocal inhibitory dilution titer; pfu = plaque-forming units; MA10 = mouse-adapted SARS-CoV-2; EPX = Eosinophil peroxidase; SD = standard deviation; LOD = limit of detection

To evaluate the impact of adjuvant formulation on the safety and efficacy of an inactivated SARS-CoV-2 vaccine (iCoV2) during homologous or heterologous viral challenge, we used established mouse models of vaccination and SARS-CoV-2 and SARS-related coronavirus (SARS-r-CoV) challenge [4, 41, 42]. BALB/c mice were vaccinated with iCoV2 derived from an early pandemic isolate [43] that was administered with either the aluminum hydroxide (Alum) adjuvant (iCoV2 + Alum) or RIBI (Sigma Adjuvant System, iCoV2 + RIBI), a research-grade adjuvant reported to induce type 1-biased immune responses [39, 40]. Mock-vaccinated mice received an irrelevant viral antigen plus Alum (inactivated influenza virus, iFLU + Alum). Both vaccine formulations were highly immunogenic as demonstrated by induction of robust neutralizing antibodies against the homologous SARS-CoV-2 strain (Fig. 1a). Next, upon challenge with pathogenic mouse-adapted SARS-CoV-2 (MA10) [41, 42], mice vaccinated with either iCoV2 formulation were protected from clinical disease compared to mock-vaccinated controls (Fig. 1b,c) and exhibited undetectable pulmonary viral titers at 5 DPI (Fig. 1d). Both vaccines also significantly reduced pulmonary pathology, as measured by acute lung injury (ALI) and diffuse alveolar damage (DAD) (Fig. 1e,f) [41, 42, 44–47]. Notably, iCoV2 + RIBI provided more complete protection than the Alum vaccine from ALI and DAD, although these differences were not statistically significant. Lastly, consistent with Dipiazza and Leist et al [37], we observed adjuvant-dependent pulmonary eosinophil infilitration and type 2 cytokine upregulation in iCoV2 + Alum-vaccinated mice (Fig. 1g,h and **Extended Data** Fig. 1a). Thus, iCoV2 is protective against pathogenic homologous virus challenge, consistent with results from inactivated COVID-19 vaccines in humans [8], but RIBI promoted improved protection from pathology, including avoidance of type 2 inflammation.

Because inactivated COVID-19 vaccines exhibit reduced efficacy against variants of concern (VOC) in humans [8, 12, 17], we further evaluated the immunogenicity of iCoV2 against a panel of VOC using an an established surrogate neutralization assay measuring inhibition of binding between human antigen-converting enzyme 2 (ACE2) and SARS-CoV-2 Spike proteins. Consistent with the real-world performance of inactivated SARS-CoV-2 vaccines, both iCoV2 formulations elicited robust neutralizing activity against early pandemic VOC, including B.1.351 (Beta) and B. 1.617.2 (Delta ) (**Extended Data** Fig. 2a-e), but little neutralizing activity against Omicron subvariants (**Extended Data** Fig. 2f-j).

We also evaluated vaccine efficacy against challenge with B.1.351 (Beta) using a mouse-adapted SARS-CoV-2 expressing the B.1.351 Spike protein (MA10-B.1.351) [48]. Similar to homologous challenge, iCoV2 induced neutralizing antibodies, promoted control of viral replication, and prevented severe clinical disease and pathology in mice challenged with MA10-B.1.351 (**Extended Data** Fig. 3a-f). Notably, we observed mild transient weight loss in vaccinated mice (**Extended Data** Fig. 3b) and mild clinical signs specifically in iCoV2 + Alum-vaccinated mice (**Extended Data** Fig. 3c). Furthermore, while iCoV2 + RIBI provided protection against respiratory pathology, iCoV2 + Alum conferred incomplete protection that was not significantly different from controls (**Extended Data** Fig. 3e,f). Lastly, consistent with the homologous challenge results, we again observed type 2 inflammation specifically associated with iCoV2 + Alum, although the magnitude of type 2 cytokine upregulation was lower than that observed during homologous challenge, which may reflect lower viral loads in this challenge model and (**Extended Data** Fig. 1b and 3g,h).

### Inactivated SARS-CoV-2 and aluminum hydroxide (iCoV2 + Alum) causes vaccine-associated enhanced respiratory disease (VAERD) during heterologous infection by a SARS-related coronavirus

**Figure 2:**
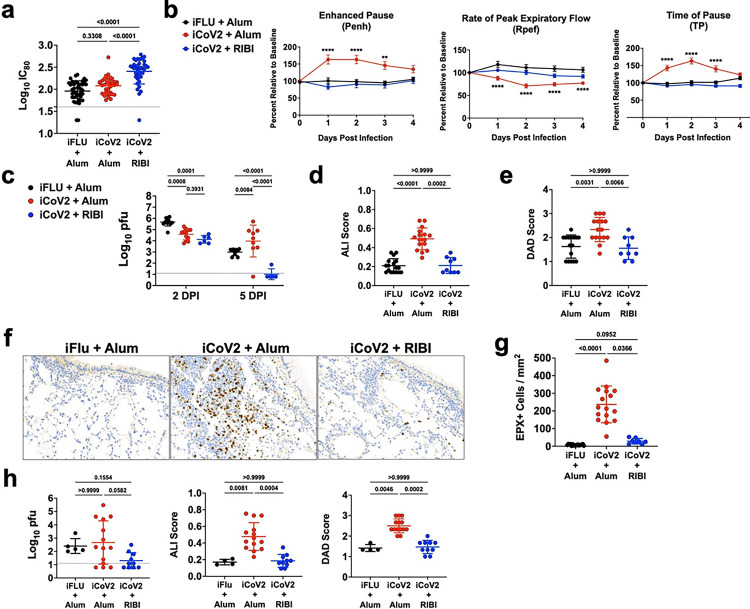
Inactivated SARS-CoV-2 + aluminum hydroxide (iCoV2 + Alum) vaccination causes enhanced subclinical disease and type 2 inflammation during infection by a SARS-related coronavirus. iFLU = inactivated influenza virus vaccine; iCoV2 = inactivated SARS-CoV-2 vaccine; Alum = aluminum hydroxide adjuvant; RIBI = Sigma Adjuvant System adjuvant; DPI = days post-infection; IC_80_ = 80% serum reciprocal inhibitory dilution titer; pfu = plaque-forming units; EPX = Eosinophil peroxidase; WBP = whole body plethysmography; SEM = standard error of the mean; SD = standard deviation; LOD = limit of detection; PenH = Enhanced Pause; Rpef = Rate of Peak Expiratory Flow; TP = Time of Pause Vaccinated mice were challenged with Rs-SHC014-CoV (SHC014) at either 4 weeks (a-g) or 10.5 months (h) post-boost. Pulmonary function was measured 1–4 DPI using WBP and pulmonary tissue was collected from vaccinated mice at 2 and 5 DPI (results displayed for 5 DPI if not specified). **(a)** Post-boost serum samples were collected prior to challenge and neutralizing antibody titers against SHC014 were measured using a luminescence-based microneutralization assay. Log-transformed results reported as IC_80_. Combined results from 4 independent replicates. **(b)** Pulmonary function of vaccinated mice was measured by whole-body plethysmography before (baseline) and during SHC014 infection. PenH, Rpef, and TP were measured 1–4 DPI. **(c)** Pulmonary viral titers at 2 and 5 DPI were quantified by plaque assay and log-transformed results reported as log_10_ pfu. **(d, e)** H&E-stained pulmonary specimens were analyzed by a blinded pathologist to evaluate ALI (d) and DAD (e). **(f, g)** Pulmonary eosinophils (EPX^+^ cells) at 5 DPI were measured using immunohistochemical staining (representative micrographs, 100X magnification in f) and quantified (g). **(h)** Pulmonary viral titers, ALI and DAD were measured in samples collected at 5 DPI following challenge at 10.5 months post-boost vaccination. **(a, c-e, g, h)** Individual data points represent biological replicates. Results analyzed by Kruskal-Wallis test with Dunn’s multiple comparisons correction (alpha = 0.05). Solid horizontal lines overlaying data represent group means. Error bars represent group SD. Dotted lines represent assay LOD. Solid horizontal lines above data represent pairwise comparisons with P values. Results from 1 (c, h), 3 (d, e, and g), or 4 (a) independent replicates (if multiple replicates, effects reproduced in each replicate). **(b)** Combined results from 2 independent replicates Reported as group mean ± SEM. Analyzed by two-way ANOVA with Tukey’s multiple comparisons (alpha = 0.05). Asterisks (*) denote P values (pairwise comparison between proximate experimental value and control iFLU + Alum value) using the following scheme: (*) = 0.01 – 0.05, (**) = 0.001 – 0.01, (***) 0.0001 – 0.001, (****) < 0.0001. Number of animals per group: iFLU + Alum (12), iCoV2 + Alum (24), iCoV2 + RIBI (12).

Preexisting vaccine-induced SARS-CoV-2 immune memory will likely impact potential future SARS-r-CoV epidemics. Based on iCoV2’s performance against VOC, we expected that iCoV2 would fail to elicit protective immunity against a heterologous pre-emergent SARS-r-CoV. To this end, we measured serum neutralization against Rs-SHC014-CoV (SHC014), a clade 3 sarbecovirus that can bind human ACE2 and replicate in human airway epithelial cells, making it a potential emerging disease threat [4]. Using mRNA vaccine sera against SARS-CoV-2 D614G, previous studies show an approximately 100-fold reduction in neutralizing titers to SHC014 [48]. Under conditions in which iCoV2 + Alum did not induce detectable neutralizing antibodies above background, iCoV2 + RIBI elicited a 3-fold increase in cross-neutralizing antibody titers against SHC014 from baseline. These responses were significantly different from both the control and iCoV2 + Alum groups (Fig. 2a), suggesting adjuvant-dependent effects on heterologous neutralizing antibody responses.

**Figure 3:**
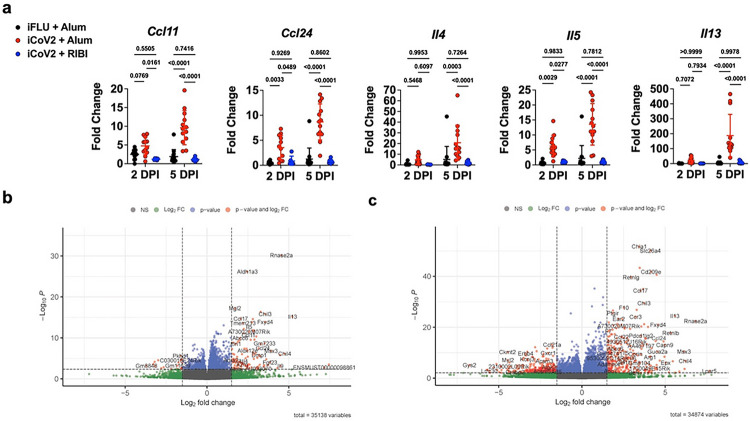
Inactivated SARS-CoV-2 vaccine (iCoV2) adjuvants promote divergent immune gene expression patterns during infection by a SARS-related coronavirus (Rs-SHC014-CoV). iFLU = inactivated influenza virus vaccine; iCoV2 = inactivated SARS-CoV-2 vaccine; Alum = aluminum hydroxide adjuvant; RIBI = Sigma Adjuvant System adjuvant; Ccl11 = C-C motif chemokine 11; Ccl24 = C-C motif chemokine 24; Il4 = Interleukin 4; Il5 = Interleukin 5; Il13 = Interleukin 13; DPI = days post-infection; SEM = standard error of the mean; SD = standard deviation; RNA-Seq = RNA Sequencing; NS = not significant; FC = fold-change Vaccinated mice were challenged with Rs-SHC014-CoV (SHC014) 4 weeks post-boost. Pulmonary tissue was collected from vaccinated mice at 2 or 5 DPI. **(a)** Type 2 cytokine gene expression from whole-tissue pulmonary specimens was measured using quantitative RT-PCR. Results reported as fold change normalized to *Gapdh* expression using DDCt. Individual data points represent biological replicates. Results analyzed by two-way ANOVA with Tukey’s multiple comparisons test (alpha = 0.05). Solid horizontal lines overlaying data represent group means. Error bars represent group SD. Solid horizontal lines above data represent pairwise comparisons with P values. Representative results from a single experiment. **(b, c)** RNA-Seq was performed with whole pulmonary tissue. Volcano plots showing differential expression between iCoV + Alum relative to iCoV + RIBI at 2 DPI (b) and 5 DPI (c) following SHC014 infection. Fold-change is shown along the X-axis (with 1.5 log_2_ fold-change thresholds represented by vertical dashed lines) and significance along the Y-axis (with FDR adjusted q < 0.05 thresholds represented by horizontal dashed lines). Key genes are highlighted in the upper right (iCoV + Alum expression > iCoV + RIBI) and upper left (iCoV + Alum expression < iCoV + RIBI) quadrants. Representative results from a single experiment.

Due to the poor cross-neutralization elicited by iCoV2 + Alum against SHC014, which increases the risk of vaccine breakthrough, we next assessed vaccine-mediated protection against SHC014 challenge. SHC014 can replicate to high levels in murine respiratory tissue but causes minimal pathology and no overt clinical disease in naïve mice [4], providing an optimal model for detecting VAERD. Consistent with this prior work, SHC014 did not cause overt clinical disease (**Extended Data** Fig. 4), alter respiratory function, or induce pathology in control mice (Fig. 2). However, iCoV2 + Alum-vaccinated mice exhibited impaired respiratory function as measured by whole body plethysmography (WBP), including altered Enhanced Pause (Penh), Rate of Peak Expiratory Flow (Rpef), and Time of Pause (TP), compared to controls (Fig. 2b). Importantly, iCoV2 + RIBI vaccination had no adverse effect on any respiratory function measure. Both vaccine formulations caused modest reductions in viral load at 2 DPI, demonstrating a degree of cross-protection (Fig. 2c). However, compared to controls, iCoV2 + Alum-vaccinated mice exhibited significantly higher viral loads at 5 DPI, with many mice exhibiting titers equivalent to those seen at 2 DPI. In contrast, iCoV2 + RIBI promoted robust clearance by 5 DPI. These results were corroborated by immunohistochemical staining of viral Nucleocapsid antigen (**Extended Data** Fig. 5a). In addition to causing impaired respiratory function and delayed viral clearance, iCoV2 + Alum vaccination caused increased pathology at 5 DPI, while iCoV2 + RIBI-vaccinated mice showed no signs of respiratory pathology (Fig. 2d,e). Furthermore, iCoV2 + Alum-vaccinated mice specifically exhibited type 2 inflammation during SHC014 infection, including increased pulmonary eosinophil infiltration by 5 DPI and type 2 cytokine expression at both 2 and 5 DPI (Fig. 2f,g and Fig. 3a). Pathological analysis also revealed additional inflammatory signs unique to iCoV2 + Alum-vaccinated mice: enhanced CD4^+^ cell infiltration at 5 DPI, C3 complement protein deposition at 2 and 5 DPI, Arginase^+^ cell infiltration at 2 and 5 DPI, and mucus production at 5 DPI (**Extended Data** Fig. 5b-e). These results clearly demonstrate that in the context of heterologous infection, vaccination with iCoV2 + Alum not only fails to protect against SHC014 replication but actually predisposes animals to enhanced virus-induced disease and delayed viral clearance. In contrast, use of a different adjuvant (RIBI) promotes viral clearance and is not associated with exacerbated disease.

We next assessed the durability of either the adverse effects of iCoV2 + Alum or the protective effects of iCoV2 + RIBI by challenging mice up to 10.5 months post-boost. We observed that iCoV2 + Alum caused delayed viral clearance by 5 DPI in a subset of mice challenged at all time points (Fig. 2h and **Extended Data** Fig. 6a), while iCoV2 + RIBI appeared to promote clearance as late as 10.5 months post-boost. WBP revealed signs of impaired respiratory function in iCoV2 + Alum-vaccinated mice challenged up to 9 months post-boost (**Extended Data** Fig. 6b). We also observed adjuvant-dependent exacerbated pathology and type 2 inflammation in mice challenged through the duration of the study (Fig. 2h and **Extended Data** Fig. 6b,c). Therefore, susceptibility to VAERD seen in iCoV2 + Alum-vaccinated animals is highly durable.

### Inactivated SARS-CoV-2 vaccine (iCoV2) adjuvants promote divergent immune gene expression patterns during heterologous infection

To systemically evaluate how iCoV2 + Alum and iCoV2 + RIBI alter the pulmonary immune environment during SHC014 infection, we conducted RNA Sequencing (RNA-Seq) and analysis of differentially-expressed genes (DEG). We found 2042 DEG at 2 DPI, 4349 DEG at 5 DPI, and 738 of these DEG at both timepoints (at a bonferonni-corrected p < 0.05). At 2 DPI, 1529 genes were significantly upregulated, and 513 were significantly downregulated, in iCoV2 + Alum mice relative to iCoV2 + RIBI counterparts (Fig. 3b). At 5 DPI, 2158 genes were significantly upregulated, and 2191 were significantly downregulated, in iCoV2 + Alum relative to iCoV2 + RIBI (Fig. 3c).

Given several of the genes that were significantly upregulated in iCoV2 + Alum-vaccinated mice were associated with type 2 inflammation and eosinophil recruitment (e.g., *Il4, Il5, Il13, Ccl11* and *Ccl24*), we sought to identify biological processes that were differentially functioning between the two vaccines. Using Gene Ontology (GO) enrichment analysis of genes that were (i) significantly DE and (ii) had a log_2_ fold-change ≥ 1.5, we found that chemokine and cytokine activity, and specifically CCR chemokine receptor binding functions, were significantly enriched in the iCoV2 + Alum group at 2 DPI (**Extended Data Table 1**). Similarly, the predominant pathways upregulated in the iCoV + Alum group at 5 DPI continued to include chemokine and cytokine signaling. In contrast, the upregulated pathways in iCoV2 + RIBI at 5 DPI showed a variety of normal processes (e.g., ion transport activities, actin and cytoskeletal binding), consistent with a resolution of infection in these animals (**Extended Data Table 1**).

### Heterologous boost vaccination partially reduces VAERD during heterologous infection

**Figure 4:**
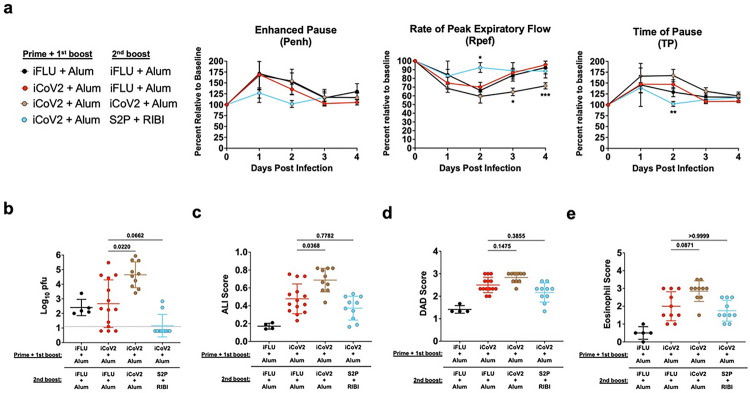
Heterologous boost vaccination partially reduces enhanced disease from vaccination with inactivated SARS-CoV-2 + aluminum hydroxide (iCoV2 + Alum). iFLU = inactivated influenza virus vaccine; iCoV2 = inactivated SARS-CoV-2 vaccine; Alum = aluminum hydroxide adjuvant; RIBI = Sigma Adjuvant System adjuvant; DPI = days post-infection; Rpef = Rate of Peak Expiratory Flow; ALI = Acute lung injury; DAD = Diffuse alveolar damage; S2P = full-length pre-fusion stabilized SARS-CoV-2 Spike protein; WBP = Whole body plethysmography Mice were initially vaccinated with inactivated SARS-CoV-2 and aluminum hydroxide adjuvant (iCoV2 + Alum). Approximately 9.5 months post-boost vaccination, mice were administered either a third dose of iCoV2 + Alum (homologous second boost) or were boosted with a heterologous vaccine formulation (recombinant full-length pre-fusion stabilized Spike protein [S2P] + RIBI) 4 weeks prior to challenge with Rs-SHC014-CoV (SHC014). Pulmonary function was measured 1–4 DPI using WBP and pulmonary tissue was collected 5 DPI. **(a)** Pulmonary function of vaccinated mice was measured by whole-body plethysmography before (baseline) and during SHC014 infection. Rpef was measured 1–4 DPI. **(b)** Pulmonary viral titers were quantified by plaque assay and log-transformed results reported as log_10_ pfu. **(c, d)** H&E-stained pulmonary specimens were analyzed by a blinded pathologist to evaluate ALI (c) and DAD (d). **(e)** Eosinophils (EPX^+^ cells) were detected using immunohistochemistry and measured using a blinded semi-quantitative eosinophil scoring system described below. **(a)** Results from a single experiment. Reported as group mean ± SEM. Analyzed by two-way ANOVA with Tukey’s multiple comparisons (alpha = 0.05). Asterisks (*) denote P values (pairwise comparison between proximate experimental value and control iFLU + Alum value) using the following scheme: (*) = 0.01 – 0.05, (**) = 0.001 – 0.01, (***) 0.0001 – 0.001, (****) < 0.0001. Number of animals per group: iFLU + Alum, iFLU + Alum (5); iCoV2 + Alum, iFLU + Alum (14), iCoV2 + Alum, iCoV2 + Alum (10); iCoV2 + Alum, S2P + RIBI (9). **(b-d)** Individual data points represent biological replicates. Results analyzed by Kruskal-Wallis test with Dunn’s multiple comparisons correction (alpha = 0.05). Solid horizontal lines overlaying data represent group means. Error bars represent group SD. Dotted lines represent assay LOD. Solid horizontal lines above data represent pairwise comparisons with P values. A sagittal section through the left lobe was scanned by a blinded analyst for eosinophils using the following 5-point scoring system: 0 = no eosinophils; 1 = rare, scattered eosinophils; 2 = small clusters of eosinophils surrounding 1 or 2 airways; 3= small to moderately sized clusters surrounding multiple airways; 4= large clusters of eosinophils surrounding multiple airways/within the parenchyma; 5= large clusters of eosinophils involving most of the lung iCoV2 + Alum – iFlu + Alum control group data are repeated from Figure 2. This group served as the iCoV2 + Alum group for the final 10.5-month post-boost vaccination (Figure 2) and the control group for comparison to later secondary boost vaccination (Figure 4).

Given the large number of people vaccinated with Alum-adjuvanted inactivated COVID-19 vaccines, the risk of individuals developing VAERD upon exposure to a heterologous coronavirus represents a potential public health risk. We therefore tested whether this risk could be ameliorated by reboosting iCoV2 + Alum-vaccinated mice with a RIBI-adjuvanted pre-fusion stabilized Spike protein vaccine (S2P + RIBI). For comparison, we also included a group that received a 3rd dose of iCoV2 + Alum. Compared to iCoV2 + Alum controls (iFLU + Alum secondary boost), a 3rd dose of iCoV2 + Alum promoted impaired respiratory function, indicated by decreased Rpef at 3 and 4 DPI (Fig. 4a), more severe delayed viral clearance (Fig. 4b), and exacerbated pathology (Fig. 4c–e and **Extended Data** Fig. 7) compared to iCoV2 + Alum controls. In contrast, secondary boost vaccination with S2P + RIBI modestly protected against impaired respiratory function, indicated by maintenance of Rpef and TP at 2 DPI, and promoted improved viral clearance, with 8 of 10 mice exhibiting no detectable virus at 5 DPI. However, reboost with S2P + RIBI did not provide significant protection against increased pathology, eosinophil infiltration, or mucus production compared to iCoV2 + Alum controls (Fig. 4c–e and **Extended Data** Fig. 7). Therefore, heterologous boost with S2P + RIBI partially protects from impaired respiratory function and delayed viral clearance, while not resolving other pathologic pulmonary responses associated with iCoV2 + Alum.

### Serum antibodies promote viral clearance while CD4^+^ T helper cells drive VAERD during heterologous infection

The strong type 2 inflammatory profile observed with iCoV + Alum-induced VAERD (Fig. 2f,g and Fig. 3) is similar to the immunopathology caused by formalin-inactivated respiratory syncytial virus (RSV) vaccination, which is reported to be mediated by type 2-biased CD4^+^ T helper (T_H_2) cells in the absence of protective antibodies [18, 19, 21–27]. Therefore, we tested the role of vaccine-induced antibodies and CD4^+^ T cells in iCoV2 + Alum-induced VAERD or iCoV2 + RIBI-mediated cross-protection.

**Figure 5:**
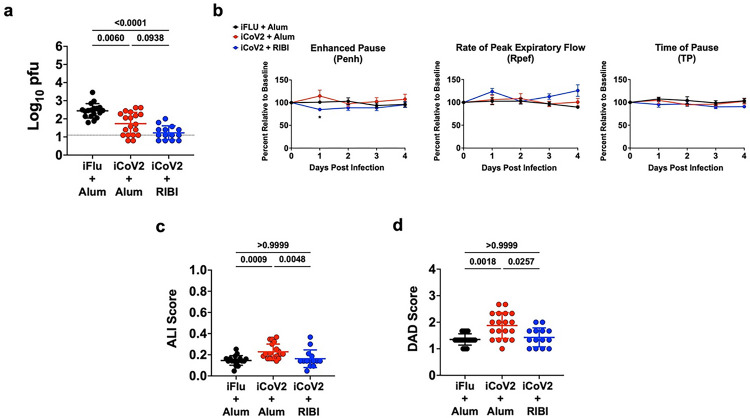
Inactivated SARS-CoV-2 (iCoV2) vaccine immune serum promotes cross-protection with modest pathology during heterologous infection. Serum was collected from mice 4-weeks post-boost vaccination and passively transferred to naïve recipient mice 1 day prior to challenge with Rs-SHC014-CoV (SHC014). Pulmonary function was measured 1–4 DPI using WBP and pulmonary tissue was collected at 5 DPI. **(a)** Pulmonary function of mice was measured by whole-body plethysmography before (baseline) and during SHC014 infection. PenH, Rpef, and TP were measured 1–4 DPI. **(b, c)** H&E-stained pulmonary specimens were analyzed by a blinded pathologist to evaluate ALI and DAD. **(d)** Pulmonary viral titers were quantified by plaque assay and log-transformed results reported as log_10_ pfu. **(a)** Results from a single experiment. Analyzed by two-way ANOVA with Tukey’s multiple comparisons test (alpha = 0.05). Data points represent group mean values. Error bars represent group Standard Error of the Mean (SEM). Asterisks (*) denote P values (pairwise comparison between proximate experimental value and control iFLU + Alum value) using the following scheme: (*) = 0.01 – 0.05, (**) = 0.001 – 0.01, (***) 0.0001 – 0.001, (****) < 0.0001. Number of animals per group: iFLU + Alum (6), iCoV2 + Alum (6), iCoV2 + RIBI (6). **(b-d)** Combined results from 2 independent replicates. Analyzed by Kruskal-Wallis test with Dunn’s multiple comparisons correction (alpha = 0.05). Solid lines represent group mean values. Error bars represent group standard deviation (SD). Individual data points represent independent biological replicates. Solid horizontal lines above data represent pairwise comparisons with P values. DPI = days post-infection; ALI = Acute lung injury; DAD = Diffuse alveolar damage; iFLU = inactivated influenza virus vaccine; iCoV2 = inactivated SARS-CoV-2 vaccine; Alum = aluminum hydroxide adjuvant; RIBI = Sigma Adjuvant System adjuvant; PenH = Enhanced Pause; Rpef = Rate of Peak Expiratory Flow; TP = Time of Pause; CD4+ dep = CD4^+^ T cell depletion; WBP = Whole body plethysmography

To test the role of antibodies in promoting viral clearance (iCoV2 + RIBI) or VAERD (iCoV2 + Alum), we used passive serum transfer followed by SHC014 challenge (**Extended Data** Fig. 8a). Consistent with our results with iCoV2 + RIBI vaccination (Fig. 2), serum from iCoV2 + RIBI-vaccinated animals promoted viral clearence compared to controls (Fig. 5a), suggesting that iCoV2 + RIBI induces cross-protective antibodies against SHC014. Somewhat surprisingly, serum transfer from iCoV2 + Alum-vaccinated mice also promoted viral clearance (Fig. 5a), indicating that iCoV2 + Alum-induced antibodies are not defective, nor do they cause antibody-dependent enhancement of infection. Further analysis found no effect of iCoV2 + Alum serum transfer on respiratory function (Fig. 5b). However, we did observe a modest but statistically significant increase in pathology in iCoV2 + Alum serum recipients (Fig. 5c,d). These results indicate that while iCoV2 + Alum vaccine-induced antibodies modestly contribute to pathology during heterologous infection, other immune system components drive iCoV2 + Alum-induced VAERD.

**Figure 6:**
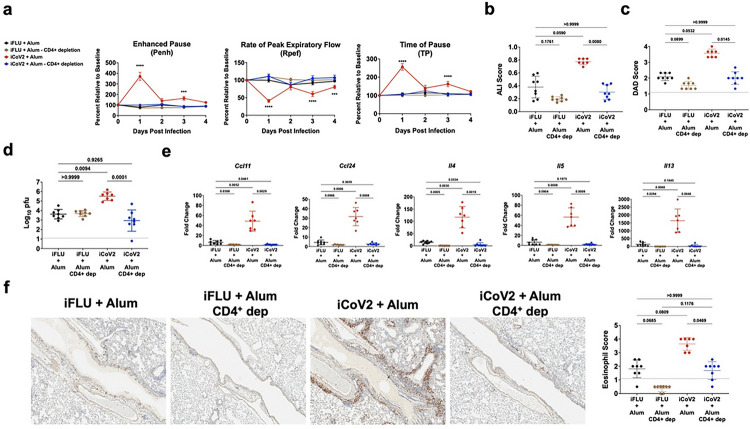
CD4^+^ T helper cells promote inactivated SARS-CoV-2 + aluminum hydroxide (iCoV2 + Alum) vaccine associated enhanced respiratory disease (VAERD) during heterologous infection. Anti-CD4 monoclonal antibody (GK1.5) was administered to mice 4-weeks post-double-boost vaccination before and during SHC014 challenge (days −5, −3 and 2 relative to challenge at day 0) to deplete CD4^+^ T helper cells (see Methods **for explanation of double-boost method).** Pulmonary function was measured 1–4 DPI using WBP and pulmonary tissue was collected at 5 DPI. **(a)** Pulmonary function of mice was measured by whole-body plethysmography before (baseline) and during SHC014 infection. PenH, Rpef, and TP were measured 1–4 DPI. **(b, c)** H&E-stained pulmonary specimens were analyzed by a blinded pathologist to evaluate ALI and DAD. **(d)** Pulmonary viral titers were quantified by plaque assay and log-transformed results reported as log_10_ pfu. **(e)** Type 2 cytokine gene expression from whole-tissue pulmonary specimens was measured using quantitative RT-PCR. Results reported as fold change normalized to *Gapdh* expression using DDCt. **(f)** Eosinophils (EPX^+^ cells) were measured using immunohistochemical staining. Representative micrographs (40X magnification) from a single experiment. **(a)** Combined results from 2 independent replicates. Analyzed by two-way ANOVA with Tukey’s multiple comparisons test (alpha = 0.05). Data points represent group mean values. Error bars represent group Standard Error of the Mean (SEM). Asterisks (*) denote P values (pairwise comparison between proximate experimental value and control iFLU + Alum value) using the following scheme: (*) = 0.01 – 0.05, (**) = 0.001 – 0.01, (***) 0.0001 – 0.001, (****) < 0.0001. Number of animals per group: iFLU + Alum control depletion (12); iFLU + Alum CD4^+^ cell depletion (12); iCoV2 + Alum control depletion (13); iCoV2 + Alum CD4^+^ cell depletion (14). **(b-f)** Results from a single experiment. Analyzed by Kruskal-Wallis test with Dunn’s multiple comparisons correction (alpha = 0.05). Solid lines represent group mean values. Error bars represent group standard deviation (SD). Individual data points represent independent biological replicates. Solid horizontal lines above data represent pairwise comparisons with P values. DPI = days post-infection; ALI = Acute lung injury; DAD = Diffuse alveolar damage; iFLU = inactivated influenza virus vaccine; iCoV2 = inactivated SARS-CoV-2 vaccine; Alum = aluminum hydroxide adjuvant; RIBI = Sigma Adjuvant System adjuvant; PenH = Enhanced Pause; Rpef = Rate of Peak Expiratory Flow; TP = Time of Pause; CD4+ dep = CD4^+^ T cell depletion; WBP = Whole body plethysmography; EPX = Eosinophil peroxidase; AB/PAS = Alcian Blue Periodic Acid-Schiff

Given that iCoV2 + Alum induced type 2 inflammation during heterologous infection (Fig. 2f,g and Fig. 3), suggesting a strong T_H_2-biased immune response, we next tested the role of CD4^+^ T cells via cell depletion in iCoV2 + Alum-vaccinated animals prior to SHC014 challenge (**Extended Data** Fig. 8b). Importantly, we observed that depletion of CD4^+^ T cells reversed all measured signs of VAERD, including impaired respiratory function, pathology, delayed viral clearance, and type 2 inflammation (Fig. 6a–g). Consistent with the hypothesis that a T_H_2-biased CD4^+^ T cell response promotes VAERD, CD4^+^ T cell depletion resulted in reversal of high type 2 cytokine expression (Fig. 6e) and significantly decreased pulmonary eosinophil infiltration (Fig. 6f). The striking impact of CD4^+^ T cell depletion on multiple adverse outcomes strongly argues that CD4^+^ T helper cells are the major drivers of iCoV2 + Alum-induced VAERD.

## DISCUSSION

Inactivated COVID-19 vaccines, of which approximately 5 billion doses have been administered, provide moderate protection against ancestral SARS-CoV-2 and early variants of concern (VOC) [7]. However, these vaccines show significantly reduced efficacy against more recent VOC [8, 12, 17]. Given that other non-protective inactivated vaccines against respiratory syncytial virus (RSV) and measles virus have been associated with vaccine-associated enhanced respiratory disease (VAERD) in both humans and animal models [18–27], we used established mouse models of homologous and heterologous viral challenge to test whether vaccine breakthrough with inactivated SARS-CoV-2 vaccines was associated with VAERD and whether this could be modulated by adjuvants. Our study demonstrates that while a model inactivated SARS-CoV-2 vaccine administered with aluminum hydroxide (iCoV2 + Alum) protected against homologous and early pandemic VOC, the vaccine caused VAERD during heterologous infection by a SARS-related coronavirus (SARS-r-CoV), Rs-SHC014-CoV (SHC014), that is normally non-pathogenic in BALB/c mice. Importantly, this outcome could be avoided (and viral clearance accelerated) by using an alternative adjuvant (RIBI, Sigma Adjuvant Systems). Although two recent reports described enhanced pulmonary pathology in preclinical animal models using SARS-CoV-2 vaccines [37, 38], to our knowledge, this is the first report to date of VAERD involving enhanced viral titers and impaired respiratory function associated with a SARS-CoV-2 vaccine. Our findings highlight the possibility that some vaccinated individuals may be placed at increased risk of VAERD due to (i) the continued evolution of more antigenically distinct SARS-CoV-2 variants, (ii) the possible emergence of heterologous SARS-r-CoVs from bat reservoirs, or (iii) the chance of re-emergence of existing SARS-CoV-2 variants from zoonotic reservoirs like deer and mink. Therefore, additional research is needed to assess the risk of VAERD in humans who have received Alum-adjuvanted inactivated COVID-19 vaccines and investigate the mechanisms underlying adjuvant-dependent protective immunity against heterologous coronaviruses. Inactivated COVID-19 vaccines, which usually include Alum [7], have an established safety and efficacy record in humans, especially against early-pandemic strains [7, 8, 12]. However, as VOC like Omicron subvariants have become the dominant circulating strains, vaccine breakthrough has become a significant problem in individuals vaccinated with inactivated vaccines [8, 12]. Importantly, these patterns were replicated in our models, in which iCoV2 + Alum protected against homologous challenge and an early-pandemic VOC but displayed breakthrough during heterologous challenge. This raises the important question of whether the immunopathology observed in this mouse model reflects a risk for humans. While enhanced type 2 immunopathology has not been reported in humans, our results suggest that adverse outcomes would not have been observed during the early stages of the pandemic, when inactivated vaccines provided substantial protection against circulating strains. However, the risk of vaccine-induced immunopathology may increase as inactivated vaccine recipients encounter more vaccine-resistant variants, such as highly divergent Omicron subvariants, or with the possible emergence of even more distinct SARS-r-CoVs in the future. Therefore, we believe that additional surveillance is needed to determine whether recipients of Alum-adjuvanted inactivated COVID-19 vaccines are at increased risk of developing adverse outcomes, with an emphasis on individuals who are predisposed to developing atopic (type 2) airway diseases.

While the direct relevance of these findings to humans remains to be determined, we believe that this work illustrates the importance of rigorously testing vaccine performance against heterologous viruses. Inactivated vaccines induce protective immunity against homologous viruses in both mouse and non-human primate studies, consistent with the performance of these vaccines during the early stages of the pandemic [7, 10, 11, 13–16, 37]. Liu et al previously evaluated an Alum-adjuvanted inactivated SARS-CoV-2 vaccine for efficacy against both SHC014 and another SARS-r-CoV, WIV1 [49] and showed partial protection against SHC014 challenge in a human ACE2 transgenic mouse model. While the differences between our work and that of Liu et al may reflect differences in the inactivation methods used for the two vaccines, or mouse-strain specific differences (as discussed below), it is also important to highlight that the Liu studies did not evaluate acute lung injury (ALI), diffuse alveolar damage (DAD), respiratory function, or pulmonary type 2 inflammation in infected animals. Therefore, our results, along with our prior work with SARS-CoV-1 vaccines [29], emphasize the importance of comprehensive analysis of coronavirus vaccines, as performed in this study, to identify adverse outcomes associated with vaccine breakthrough.

The disease signs and pulmonary pathology observed in iCoV2 + Alum-vaccinated mice during SHC014 infection are strikingly similar to phenotypes observed in models of RSV VAERD, including increased pulmonary eosinophil infiltration, inflammatory damage, and impaired respiratory function [18–27]. These prior studies with RSV demonstrated that the inactivation method used to generate the vaccine destroyed protective epitopes, causing vaccine breakthrough and VAERD driven by immune responses against non-protective epitopes. The inactivated SARS-CoV-2 vaccine used in this study elicited protective immunity against mouse-adapted viruses based on ancestral SARS-CoV-2 (SARS-CoV-2 MA10) and the B.1.351 variant (MA10-B.1.351), while iCoV2 + RIBI promoted clearance of SHC014, which argues against a loss of protective epitopes. Instead, we believe that the antigenic mismatch between the vaccine and SHC014, in combination with the type 2-biased immune response associated with Alum, is the primary cause of vaccine failure and VAERD. While passive transfer of serum from iCoV2 + Alum-vaccinated mice induced modest immunopathology upon SHC014 challenge, CD4^+^ T cell depletion completely ameliorated the delayed viral clearance and pulmonary pathology in SHC014-infected mice. This suggests that CD4^+^ T cell epitopes conserved between SARS-CoV-2 and SHC014 are the major determinants of VAERD. Therefore, to assess whether VAERD is unique to inactivated SARS-CoV-2 vaccines or is a potential outcome with other vaccine platforms, it will be important to determine whether these adverse responses are driven by epitopes within the Spike protein, which is present in all SARS-CoV-2 vaccines, or by epitopes within the highly conserved Nucleocapsid, Envelope, and Membrane proteins present in whole virus-based inactivated vaccines [7].

As discussed above, our observations are reminiscent of VAERD caused by the failed formalin-inactivated RSV vaccine tested in the 1960s, which caused exuberant type 2 inflammation and eosinophilic/neutrophilic pulmonary infiltrates in vaccinated subjects [18–27]. Given the type 2 cytokine response observed in iCoV2 + Alum-vaccinated animals and the fact that CD4^+^ T cells are critical to the adverse vaccine responses, we hypothesize that TH2s infiltrate infected pulmonary tissue and drive type 2 inflammation via secretion of cytokines like interleukin (IL)-4, IL-5 and IL-13. This suggests that anti-atopic therapies targeting these cytokines may be beneficial should VAERD be observed in humans. Our results also raise the question of whether it is possible to reprogram type 2-biased responses elicited by iCoV2 + Alum and thereby reduce the risk of VAERD. Despite potential biological limitations, the practicality of this approach is supported by its similarity to current real-world vaccination strategies [8, 50–55]. Our observation that follow-up boost vaccination with pre-fusion stabilized Spike protein (S2P) + RIBI partially reduced VAERD in mice initially vaccinated with iCoV2 + Alum suggests that this may be a promising strategy to reduce or prevent VAERD. Likewise, it will be important to determine whether infection with SARS-CoV-2 resets inactivated COVID-19 vaccine + Alum-induced type 2 immunity, as this would have important implications for the long-term risk for inactivated vaccine recipients should a novel heterologous SARS-r-CoV emerge in a vaccinated population.

The BALB/c model used in this study is predisposed to strong type 2-biased immune responses [56, 57], which likely increases susceptibility to type 2 VAERD. This has important implications for individuals vaccinated with Alum-adjuvanted inactivated vaccines who may be at risk of adverse outcomes, particularly individuals predisposed to atopic immune responses. However, prior work with coronavirus vaccines has demonstrated that vaccination can induce enhanced pulmonary eosinophil infiltration following viral challenge in both BALB/c and C57BL/6 mice and cause VAERD in other animal models [21, 27–34, 36–38]. Therefore, future studies should investigate the impact of host genetic factors on VAERD risk, and whether specific human populations are at increased risk of VAERD.

This study demonstrates that adjuvant choice is critical to optimizing vaccine design. Our results indicate that Alum, which is often reported to promote type 2 immune responses in preclinical models [19, 58–60], is a major determinant of VAERD in this model and suggests that inactivated vaccines formulated with Alum may exhibit a suboptimal safety profile in the context of heterologous infections. In contrast, our finding that using RIBI averts VAERD and even promotes more efficient viral clearance has potentially important implications for universal coronavirus vaccine design including adjuvant selection. Although it remains unclear what immune system components induced by iCoV2 + RIBI vaccination are responsible for heterologous protection, we found that passive serum transfer from vaccinated mice to naïve recipients resulted in significantly enhanced clearance of SHC014. We also observed a modest induction of SHC014-specific neutralizing antibody by iCOV2 + RIBI vaccination. However, we and others have also demonstrated that non-neutralizing antibodies can mediate cross protection during coronavirus infection [61, 62]. Therefore, studies are underway to resolve the relative contribution of neutralizing and non-neutralizing antibodies, as well as cellular immunity against conserved epitopes, in mediating cross-protection.

In summary, our findings highlight the potential risk of inactivated SARS-CoV-2 vaccine-induced immunity in the context of infections by both SARS-CoV-2 VOC and highly heterologous SARS-r-CoVs. Unlike the COVID-19 pandemic, possible future coronavirus epidemics will occur in the context of widespread pre-existing SARS-CoV-2 immunity acquired through infection and/or vaccination. This critical new variable is almost certain to impact the course of future SARS-r-CoV epidemics and should therefore be incorporated into pandemic preparedness strategies. This model anticipates continued SARS-CoV-2 variant evolution and future SARS-r-CoV emergence as important considerations in assessing vaccine safety and efficacy and represents a reproducible approach to predictively model such variables. By elucidating factors that drive beneficial or harmful cross-reactive vaccine-induced immune responses during heterologous infection, this investigation advances the development of safe and effective vaccination strategies that will increase SARS-r-CoV pandemic preparedness while mitigating potential adverse outcomes like VAERD. These findings are also useful for the development of pan-coronavirus vaccines and inform the potential utility of authorized SARS-CoV-2 vaccines during the early stages of future SARS-r-CoV epidemics.

## METHODS

### Viruses

icSARS-CoV-2 wild-type [43], mouse-adapted MA10 [41, 42], mouse-adapted MA10 expressing SARS-CoV-2 B.1.351 [48] and SHC014 [4] as well as reporter viruses SARS-CoV-2-nLuc [41, 42], B.1.351-nLuc [48] and SHC014-nLuc [48, 63] were cultured on USAMRIID Vero E6 cells in Dulbeccos Modified Eagle’s Medium (DMEM) containing 5% heat-inactivated FBS (HI-FBS). All virus work was performed in a biosafety level (BSL) 3 laboratory by personnel wearing appropriate personal protective equipment (PPE), including Powered Air Purifying Respirators (PAPRs), according to the guidelines outlined in the 6th edition of *Biosafety in Microbiological and Biomedical Laboratories (BMBL)*.

### Vaccines

Inactivated SARS-CoV-2 vaccine (iCoV2) was produced as previously published using the icSARS-CoV-2 wild-type strain [43] following the method of Spruth et al [64]. Culture supernatants from Vero E6 USAMRIID cells seeded with wild-type SARS-CoV-2 were collected and centrifuged to remove cell debris. The resulting clarified supernatant was treated with 0.05% formalin for 48 hours at 4°C. The formalin inactivated virus was exposed to 25mJ UV light, then placed in a polyallomer ultracentrifuge tube and underlayed with 20% sucrose, and then centrifuged at 24,000rpm overnight. The pellet containing the purified inactivated virus (iCoV2) was recovered in PBS and frozen at −80°C until use. For each vaccination, vaccine was mixed with adjuvant per manufacturers protocol resulting in the delivery of 0.2 μg of adjuvanted vaccine in 10 μL volume. Inactivated A/PR8 (Charles River Laboratory) influenza was prepared with adjuvant following the adjuvant manufacturer’s protocol with a final inactivated vaccine dose of 0.2 μg in 10 μL volume delivered to the left rear footpad. Adjuvants were Alhydrogel (Aluminum hydroxide/Alum, Invivogen) and RIBI (Sigma Adjuvant System, Sigma).

### Mouse vaccination and challenge model

All mouse studies were conducted under protocols approved by the Institutional Animal Care and Use Committee (IACUC) at the University of North Carolina at Chapel Hill, an AAALAC International-accredited institution, in alignment with the recommendations outlined in the *Guide for the Care and Use of Laboratory Animals*. Young adult (6–8 weeks old) female BALB/cAnNHsd mice (Envigo/Inotiv) were lightly anesthetized with isoflurane and vaccinated with 0.2 μg of inactivated SARS-CoV-2 (iCoV2) or inactivated influenza virus (iFLU, mock-vaccinated) delivered in a 10 μL volume into the left rear footpad. In selected experiments (**Extended Data 1 and 3**), phosphate-buffered saline (PBS) alone was used for mock-vaccination. Mice were boosted with 0.2 μg of vaccine three weeks post-initial vaccination. Submandibular bleeds were collected pre-prime, 3 weeks post-prime and 3 weeks post-boost. Approximately three weeks post-boost, vaccinated and boosted mice were lightly anesthetized with 50 mg/kg ketamine + 5 mg/kg xylazine and challenged intranasally with 50 μL of MA10 (1 × 10^4^ PFU), MA10-B.1.351 (5 × 10^4^ PFU) or Rs-SHC014-CoV (SHC014) (1 × 10^5^ PFU), or mock challenged with 50 μL of PBS alone. Post-challenge, mice were monitored, weighed, and scored for clinical signs daily. Mice were euthanized 2 or 5 days post infection (DPI) unless a mouse reached humane endpoint criteria (clinical score of 4 or higher) before 5 DPI. The clinical scoring scheme is as follows: 0 = clinically normal; 1 = piloerection; 2 = piloerection and kyphosis; 3 = piloerection, kyphosis, and reduced movement; 4 = markedly reduced movement and/or dyspnea; 5 = moribund, dead, or euthanized [65]. Mice were euthanized by an overdose of isoflurane anesthesia (Baxter), blood was collected by cardiocentesis, and tissues were collected for post-mortem analysis.

### Multiplex ACE2 inhibition assay

SARS-CoV-2 Spike, along with circulating variants, such as Alpha, Beta, Delta, and Omicron subvariants in the multiplexed Meso Scale Discovery (MSD) V-PLEX SARS-CoV-2 Panel-25, were used to measure ACE2 blocking antibodies. Briefly, 96-well plates were blocked using MSD Blocker A for 30 minutes and washed. Vaccinated and mock-vaccinated control serum samples (diluted 1:50) and calibrator standards were added to the plate and incubated for 1 hour at 22°C, shaking at 700 RPM. After incubation, MSD SULFO-tagged Human ACE2 was added to the wells for detection, incubated at 22°C for 1 hour, and then washed. The plate was read on the MESO QuickPlex SQ 120 instrument, and ACE2 blocking activity was analyzed using the equation: ([1 – Average Sample ECL Signal / Average ECL signal of the blank well] × 100).

### Pathology

Left lung lobes of mice were collected at necropsy, infiltrated with 100 μL 10% neutral buffered formalin (NBF), and then immersion fixed in 10% NBF for 7 days. After transfer of the fixed lung lobes to a new tube of 10% formalin, the lungs were removed from the BSL3 lab for preparation for histology submission. Lungs were rinsed with PBS (Gibco), placed in cassettes, and stored in 70% ethanol until the tissue was paraffin embedded and sectioned. Specimens were processed on an automated tissue processor (Leica ASP 6025), embedded in paraffin (Leica Paraplast), sectioned at 5 μm thickness, and stained with hematoxylin and eosin (H&E, Richard Allan Scientific). For embedding, lungs were placed in a standardized orientation to best visualize the main bronchus.

Lung histopathology was evaluated and scored by an ACVP-board certified veterinary pathologist on H&E-stained sections. Lung pathology was quantified using two scoring systems previously validated for respiratory coronavirus infection in mice [41, 42, 66] with the pathologist blinded to the status of the study groups. Three representative alveolar high power fields (HPF; 400X total magnification) were selected per H&E tissue section and scored using previously published semi-quantitative acute lung injury (ALI) and diffuse alveolar damage (DAD) scoring systems [44–47]. Briefly, ALI scores were determined as follows: (A) neutrophils in alveolar spaces (none = 0, 1–5 cells = 1, > 5 cells = 2); (B) neutrophils in alveolar septae (none = 0, 1–5 cells = 1, > 5 cells = 2); (C) well-formed hyaline membranes (none = 0, one membrane = 1; > 1 membrane = 2); (D) proteinaceous material/debris in air spaces (none = 0, one area = 1, > 1 area = 2); (E) alveolar septal thickening (> 2x mock animal thickness = 0, 2–4x mock thickness = 1, > 4x mock thickness = 2). ALI scores were calculated as follows: [(20 × A) + (14 × B) + (7 × C) + (7 × D) + (2 × E)] / 100. DAD scores were determined as follows: 1 = within normal limits; absence of cellular degeneration, sloughing, and necrosis; 2 = uncommon solitary cell sloughing and necrosis, 3 or less foci per HPF; 3 = multifocal (more than 3 foci per HPF) cellular degeneration, sloughing and necrosis +/− septal wall hyalinization/early hyaline membrane formation; 4 = severe (> 75% of field) cellular degeneration and sloughing with prominent necrosis, or the presence of at least one well-formed hyaline membrane. For each ALI and DAD scores, average of scores from three representative fields per tissue section determined final score for the specimen.

### Immunohistochemistry

Formalin-fixed tissues were processed on a Leica ASP 6025 tissue processer, embedded in paraffin (Leica Paraplast) and sectioned at 5 μm thickness onto positively charged slides. Sequential tissue sections were labeled for antigens using anti-CD4 antibody (ab183685, Abcam), anti-C3 Complement antibody (55444, MP Biomedical), or Arginase-1 (93668S, Cell Signaling Technology) on the Ventana Discovery automated staining platform (Roche), or with anti-SARS Nucleocapsid antibody (NB100–56576, Novus Biological) or anti-EPX antibody (PA5–62200, Invitrogen) on the Bond III (Leica Biosystems) automated stainer. Briefly, for labeling performed in the Ventana Discovery platform, antigen retrieval was accomplished using CC1 pH 8.5 (950 − 500, Roche) or Protease 2 (760–2019, Roche). After pretreatment, tissues were blocked, and then incubated with either anti-CD4 antibody at 1:1000, anti-C3 Complement antibody at 1:100, and Arginase-1 antibody at 1:100 for 1 hour. Secondary antibodies used were Discovery OmniMap anti-Rabbit HRP (760–4311, Roche) or Dako’s anti-Goat HRP (P0160), followed by stain development with Discovery Chromo Maps DAB (760 − 159, Roche) and Hematoxylin II (790–2208, Roche) for nuclear staining. For labeling performed on the Bond platform, slides were dewaxed in Bond Dewax solution (AR9222) and hydrated in Bond Wash solution (AR9590) from Leica Biosystems. Heat-induced antigen retrieval was performed at 100°C in either Bond-Epitope Retrieval solution 1 pH-6.0 (AR9961, Leica) or Bond-Epitope Retrieval solution 2 pH-9.0 (AR9640, Leica). After pretreatment, tissues were blocked, and then incubated with either anti-EPX antibody at 1:1000 or anti-SARS Nucleocapsid antibody at 1:8000 dilution for 1 hour followed with Leica’s Novolink Polymer (RE7260-K) secondary antibody. Antibody detection with 3,3’-diaminobenzidine (DAB) was performed using the Bond Intense R detection system (DS9263, Leica). Stained slides were dehydrated and coverslipped with Cytoseal 60 (23–244256, Thermo Fisher Scientific). A positive control was included for each run of this assay.

For image analysis, a composite image composed of 5X5 fields at 200X magnification of the inferior section of a single section of left lung was collected (3.53–3.57 mm^2^ total area). The same region of each lung was imaged and was centered around the main bronchus. Image analysis was performed using Nikon Elements software. A threshold for positive staining was set, and thresholds for area, circularity, equal diameter were set for positive object count. Results were reported as the number of EPX positive cells per mm^2^.

For semi-quantitative eosinophil scoring, a sagittal section through the entire left lung lobe was scanned by a blinded analyst for eosinophils using the following 5-point scoring system: 0 = no eosinophils; 1 = rare, scattered eosinophils; 2 = small clusters of eosinophils surrounding 1 or 2 airways; 3 = small to moderately sized clusters surrounding multiple airways; 4 = large clusters of eosinophils surrounding multiple airways/within the parenchyma; 5 = large clusters of eosinophils involving most of the lung.

### Alcian Blue Periodic Acid-Schiff (AB/PAS)

AB/PAS stains were used to identify mucus in pulmonary airways and were performed using the Leica Autostainer XL. Samples were first baked at 60 degrees Celsius for 60 minutes minimum and deparaffinized in xylene and hydrated with graded ethanols. Tissue sections were then stained with Alcian Blue-Periodic Acid Schiff (AB/PAS) using the Leica Autostainer XL. The slides were stained with Alcian Blue (867, Anatech, LTD) for 10 minutes, immersed in Periodic Acid (A223–100, Thermo Fisher Scientific) for 5 minutes, rinsed in water, then transferred to Schiff reagent (SS32–500, Fisher Scientific) for 30 minutes followed by a Sulfurous rinse for 1 minute, and finally washed in running tap water for 10 minutes. After staining, slides were then dehydrated and coverslipped with Cytoseal 60 (23–244256, Thermo Fisher Scientific). A positive control slide was included for each run of this assay.

### Viral titers

At necropsy, the superior and middle lung lobes were collected and frozen in a tube containing DMEM (Gibco) with 5% heat-inactivated FBS with glass beads and frozen at −80 degrees until the day of titer assay. At time of assay, tissues were thawed, homogenized for 40 seconds at 6,000 rpm using a MagNA Lyser (Roche) and centrifuged to clarify the sample from residual tissue and beads. A 50 μL sample aliquot of clarified homogenate was removed and added to 450 μL of DMEM + 5%HI-FBS. The resulting dilution was used to make additional 10-fold dilutions. An aliquot (200 μL) of each dilution was plated in duplicate in 12 well plates containing Vero E6 USAMRIID monolayers. Plates were gently rocked every 15 minutes to ensure uniform distribution of virus across the monolayer. After one hour of rocking, carboxymethylcellulose (CMC) overlay (2.5% CMC and 1x-alpha MEM) was added to each and the plates were incubated at 37°C for 4 days, at which point plates were fixed with 4% paraformaldehyde overnight. After fixation, fix/overlay was removed, cell monolayers were stained with 0.25% crystal violet and plaques were counted.

### Quantitative RT-PCR

At necropsy, postcaval lung lobes were collected for RNA isolation. Lung lobes were placed in TRIzol (Invitrogen) with glass beads and homogenized at 6,000rpm for 40 seconds using a MagNA Lyser homogenizer. The homogenates were centrifuged to clarify the homogenate from tissue debris and beads. Clarified samples were transferred to a second tube and removed from the BSL3 lab. RNA was isolated from the sample using the Qiagen RNeasy Kit (Qiagen) following the manufacturer’s protocol. Using the High-Capacity cDNA reverse transcription kit (Applied Biosystems), cDNA was synthesized by adding 1 μg of RNA per reverse transcription reaction. Quantitative PCR was performed for each of the primer-probe sets using the TaqMan Fast Advanced Master Mix (Applied Biosystems). Two μL of cDNA were added per reaction. All genes were normalized to *Gapdh* expression and reported as DDCt. Limit of detection was determined by water controls and set for all primers at a Ct of 34.

Primer-probe sets used:

*Gaphd* (Applied Bioystems, Catalogue # 4352339E, Probe VIC/MGB);

*Ccl11* (Integrated DNA Technologies, Catalogue # Mm.PT.58.28587819, Probe FAM/ZEN/IBFQ);

*Ccl24* (Integrated DNA Technologies, Catalogue # Mm.PT.58.13396581, Probe FAM/ZEN/IBFQ);

*Il4* (Integrated DNA Technologies, Catalogue # Mm.PT.58.7882098, Probe FAM/ZEN/IBFQ);

*Il5* (Integrated DNA Technologies, Catalogue # Mm.PT.58.41498972, Probe FAM/ZEN/IBFQ);

*Il13* (Integrated DNA Technologies, Catalogue # Mm.PT.58.31366752, Probe FAM/ZEN/IBFQ).

### Neutralization assay

Post-boost serum samples from vaccinated mice were evaluated for neutralizing antibody levels using an established SARS-CoV-2 neutralization assay [43, 48, 63]. Blood was collected from vaccinated mice 19–21 days post-boost vaccination and centrifuged at 5000 × g for 5 min. Immune serum samples were heat inactivated at 56°C for 30 min and three-fold serial dilutions were mixed with equal amounts of diluted icSARS-CoV-2-nLuc (D614G) [43], icSARS-CoV-2-B.1.351-Spike-nLuc [48], or icSHC014-CoV-nLuc [48, 63] incubated at 37°C with 5% CO_2_ for one hour. Following incubation, virus-sera mixtures were added to duplicate wells in a 96 well dish containing Vero E6 C1008 cells and incubated for 24 hours. Virus-only controls as well as cell-only controls were included in each neutralization assay plate. After a 24-hour incubation, cells were lysed, and luciferase activity was measured via Nano-Glo Luciferase Assay System (Promega) according to the manufacturer’s protocol. Neutralization titers were defined as the sample dilution at which 80% reduction in relative light unit (RLU) was observed relative to the average of the control wells (80% reciprocal inhibitory concentration [IC80]). For the icSHC014-CoV-nLuc assay, normal mouse serum shows high background neutralizing activity against SHC014, which reduced the sensitivity of the assay

### Whole body plethysmography (WBP)

WBP measurement was performed using DSI Finepointe WBP hardware and analyzed using DSI Finepointe Software as previously described [67]. Measurements were acquired for individual mice using the COPD study type and WBP Volume apparatus at baseline and once each day, 1 through 4 days post-infection (DPI) (i.e., measurements included: Baseline, 1 DPI, 2 DPI, 3 DPI, and 4 DPI). Reported measurements are the average parameter value measured during a 10-minute data acquisition period that followed a 20-minute acclimation period. 1–4 DPI measurements were reported as percentage relative to baseline measurement.

### Passive serum transfer

Donor BALB/c mice were vaccinated using the prime-boost method with either iFlu + Alum, iCoV2 + Alum, or iCoV2 + RIBI (see ‘Mouse vaccination and challenge model’). At 4 weeks post-boost vaccination, blood was collected from sacrificed donor mice via cardiac puncture. Individual blood samples were incubated for at least 30 minutes to allow clotting and centrifuged at 5000 × g for 5 minutes for serum separation. Equal volumes of individual serum samples were pooled within each vaccine. Approximately 225 μL of fresh pooled immune serum was transferred to appropriate age-matched naïve recipient mice via intraperitoneal injection 1 day prior to challenge with Rs-SHC014-CoV (SHC014).

### CD4^+^ T cell depletion

BALB/c mice were vaccinated using a double-boost method with either iFlu + Alum or iCoV2 + Alum. To increase the power of individual experiments, after vaccination using the prime-boost method (see ‘Mouse vaccination and challenge model’), mice were subsequently administered a third dose of the same vaccine (see Fig. 4; iCoV2 + Alum double-boost group exhibits higher magnitude and lower variability in some parameters of iCoV2 vaccine-associated enhanced respiratory disease [VAERD]). At 4 weeks post-2nd boost vaccination, anti-CD4 monoclonal antibody (GK1.5, BioXCell BE0003–1) or isotype control monoclonal antibody (LTF-2, BioXCell BE0090) were administered to mice via intraperitoneal injection in 250 μL of phosphate-buffered saline at day − 5 (500 μg per mouse), day − 3 (250 μg per mouse), and day 2 (125 μg per mouse) relative to challenge at day 0 with Rs-SCH014-CoV (SHC014).

### RNA-Seq

RNA was extracted as described above. Quality (RIN and DV200) was assessed via Tapestation (Agilent, Santa Clara, CA). Samples with a RIN < 2 were excluded from moving forward. Libraries for RNAseq were generated at the UNC High Throughput Sequencing Facility with a Kapa total RNA stranded library prep with Ribo Erase. Samples were barcoded and pooled before running on an Illumina NovaSeq (whole S4 flowcell). We generated 1×50 SE reads at a median coverage of 115.47 million reads (Range: 69.25–185.95 million reads). We ran FastQC (v0.11.9) [68] to confirm data quality, with all samples passing. Following QC, we ran Salmon (v1.10.0) [69] to quantify transcripts. We used alignment based mode, adjusting for GC bias in reads, and bootstrapping with inferential replicates. Following quantification, we imported gene-level count matrices using *tximport* (v1.28.0) [70] for use in differential expression analyses. We normalized count data with DESeq1 (v1.40.2) [71], and generated logarithmic fold changes and metrics of significance (raw and adjusted p-values, false discovery rate q-statistics). Differentially expressed genes (DEG) with an adjusted p-value of < 0.05 were run through Gene Ontology (GO) analyses via PANTHER 17.0 [72–74]. All analyses were run in Bioconductor and the R statistical package.

### Statistics

Statistical analyses were performed using Graphpad Prism 9. Evaluation for differences between group means were evaluated using (i) Kruskal-Wallis with Dunn’s multiple comparisons test (when data did not fulfill criteria for assumption of Gaussian distribution), (ii) Brown-Forsythe and Welch’s ANOVA with Dunnett T3 correction (when data fulfilled criteria for assumption of Gaussian distribution but not for assumption of equal Standard Deviation), or (iii) ordinary two-way ANOVA with Tukey’s multiple comparisons test (for grouped analyses). Specific comparisons evaluated for each data set are specified in figures. Statistical tests are corrected for multiple comparisons and/or repeated measures. For all statistical analyses, multiplicity adjusted p-values (two-tailed when applicable) were computed using an alpha threshold of 0.05. When appropriate, data are presented as scatter dot plots showing individual data points with group mean values represented using a horizontal line. Two-sided error bars represent Standard Deviation or Standard Error of the Mean (specified in figure legends). When possible, p-values are represented numerically in data figures showing pairwise comparisons. Otherwise, asterisks (*) are used to denote p-values using the following scheme: (*) = 0.01–0.05, (**) = 0.001–0.01, (***) 0.0001– 0.001, (****) < 0.0001

## Data Availability

Raw data and information required for reanalysis are available from the corresponding authors upon request or will be submitted to a publically available database. RNA Sequencing raw .fastq data are submitted to the Sequence Read Archive (SRA) database under BioProject ID PRJNA1022427.
